# Biofilm and Planktonic Antibiotic Resistance in Patients With Acute Exacerbation of Chronic Rhinosinusitis

**DOI:** 10.3389/fcimb.2021.813076

**Published:** 2022-01-17

**Authors:** Henrique Augusto Cantareira Sabino, Fabiana Cardoso Pereira Valera, Denise Vieira Santos, Marina Zilio Fantucci, Carolina Carneiro Titoneli, Roberto Martinez, Wilma T. Anselmo-Lima, Edwin Tamashiro

**Affiliations:** ^1^ Department of Ophthalmology, Otorhinolaryngology, and Head and Neck Surgery, Division of Otorhinolaryngology, Ribeirão Preto Medical School, University of São Paulo, Ribeirão Preto, Brazil; ^2^ Department of Internal Medicine, Division of Infectious Diseases, Ribeirão Preto Medical School, University of São Paulo, Ribeirão Preto, Brazil

**Keywords:** chronic rhinosinusitis, disease exacerbation, antimicrobial susceptibility, biofilm, microbial susceptibility tests, antibiofilm, minimum inhibiting and bactericidal concentrations

## Abstract

**Introduction:**

The recalcitrant nature of patients with acute exacerbation of chronic rhinosinusitis (AECRS) potentially involves persisting colonization of the sinonasal mucosa by bacterial biofilms. Biofilms are known to be highly resistant to antibiotics, which may trigger or maintain chronic inflammation in the sinonasal mucosa. However, little is known about the relationship between the minimum inhibitory concentration (MIC) and antibiofilm concentrations of bacteria obtained from AECRS patients.

**Material and Methods:**

Thirty bacterial strains from 25 patients with AECRS were identified and underwent MIC determination (VITEK^®^ 2). The planktonic isolates were submitted to an *in vitro* formation of biofilms (Modified Calgary Biofilm Device) and determination of minimum biofilm inhibitory concentration (MBIC) and minimum biofilm eradication concentration (MBEC) for amoxicillin, amoxicillin/clavulanic acid, clarithromycin, and levofloxacin. MIC of the planktonic forms was compared with MBIC and MBEC levels, according to the breakpoints established by the Clinical Laboratory Standards Institute guidelines.

**Results:**

The main bacteria retrieved was *S. aureus* (60%), followed by other Gram-positive and Gram-negative bacteria in lower frequencies. 76.7% of strains formed biofilm *in vitro* (n=23/30). The planktonic isolates presented high rates of resistance for amoxicillin (82.6%) and clarithromycin (39.1%), and lower rates for amoxicillin/clavulanic acid (17.4%). The biofilm-forming bacteria counterparts presented higher levels of MBIC and MBEC compared to the MIC levels for amoxicillin, amoxicillin/clavulanic acid, and clarithromycin. Levofloxacin was highly effective against both planktonic and biofilm forms. Planktonic resistant forms were associated with levels of antibiofilm concentrations (MBIC and MBEC).

**Conclusions:**

Biofilm-forming bacteria from AECRS patients are prevalent, and biofilm forms are highly resistant to antibiotics compared to their planktonic counterparts. Antibiotic resistance observed in planktonic forms is a good indicator of biofilm resistance, although near 20% of susceptible planktonic bacteria can produce antibiotic tolerant biofilms.

## Introduction

The potential role of bacteria in the pathogenesis of chronic rhinosinusitis (CRS) involves multiple facets of living bacteria, including intracellular cells, free-floating planktonic bacteria, and biofilm attached to the sinonasal mucosa (Lam et al., 2015; Maina et al., 2018; Vestby et al., 2020). Bacterial biofilms, a sessile and a ubiquitous form in the bacterial life cycle, are broadly found in the sinonasal mucosa of CRS patients in 44-92% of cases, depending on the method used for detection (Zhao and Wormald, 2017; Hamilos, 2019).

In chronically infected CRS patients, reducing or eliminating the pathogenic bacterial burden may ameliorate the sinonasal inflammation, with substantial medical improvement. However, most antimicrobial therapy directed against these sinonasal pathogens is based on the planktonic bacteria susceptibility *in vitro*, which may underestimate the more resistant forms of bacteria. Biofilms, for instance, have been reported to present a 100-1,000-fold increase tolerance relative to the planktonic cell counterparts (Ceri et al., 1999; Yan and Bassler, 2019), caused by a multitude of distinct mechanisms.

To date, few studies have investigated the biofilm resistance profile in CRS patients for specific antibiotics, such as amoxicillin/clavulanic acid, macrolides, quinolones, and mupirocin (Desrosiers et al., 2007; Ha et al., 2008; Božić et al., 2018). To the best of our knowledge, no studies have investigated the relationship between planktonic and biofilm resistance in patients with CRS. As patients with acute exacerbation of CRS (AECRS) are potentially a surrogate of a biofilm-related infection paradigm (Szaleniec et al., 2019), we chose this clinical condition to explore the relationship between the minimum inhibitory concentration (MIC) and antibiofilm concentrations.

## Material and Methods

### Patient Selection

Adult patients (>18 years old) with AECRS were selected in a tertiary rhinology clinic (Clinics Hospital of the Ribeirão Preto Medical School, Brazil) between January 2012 and January 2014. CRS was established according to the EPOS 2012 criteria, which included persisting sinonasal symptoms lasting for more than 12 weeks (nasal obstruction/congestion or nasal secretion should be present), with sinonasal inflammatory signs present at computed tomography or nasal endoscopy. Acute exacerbation of CRS was defined as an acute worsening of sinonasal symptoms in the last four weeks (nasal secretion, nasal obstruction/congestion, sense of smell, and/or facial pain) in patients with underlying CRS (Fokkens et al., 2012). We excluded from the study patients who had received antibiotics orally or topically in the last 30 days, patients under suspicion or confirmed immunodeficiency, primary ciliary dyskinesia, cystic fibrosis, allergic fungal rhinosinusitis, benign or malignant sinonasal tumors.

### Planktonic Bacteria Assays

A swab from the middle meatus was collected guided by nasal endoscopy and was seeded on agar plates (sheep blood, MacConkey, and mannitol salt) and incubated at 37°C for 24 hours for microbial identification in the automated VITEK^®^ device (BioMérieux). Complementary tests were performed to characterize genus and species whenever necessary. For planktonic bacteria, the antimicrobial susceptibility profile was determined by the VITEK^®^ 2 card system (BioMérieux, AST-P612, AST-GN), as well as minimum inhibitory concentration (MIC) was determined by the E-test^®^ method (BioMérieux) for the following antibiotics: amoxicillin, amoxicillin/clavulanic acid (AMX-CLAV), clarithromycin, and levofloxacin. The determination of MIC breakpoints followed the guidelines of the Clinical Laboratory Standards Institute (CLSI) (CLSI, 2018). Bacterial strains from positive cultures were stocked at -70°C in tryptic soy broth with 20% glycerol until further testing involving biofilms.

### Biofilm Bacteria Assays

**Figure 1:**
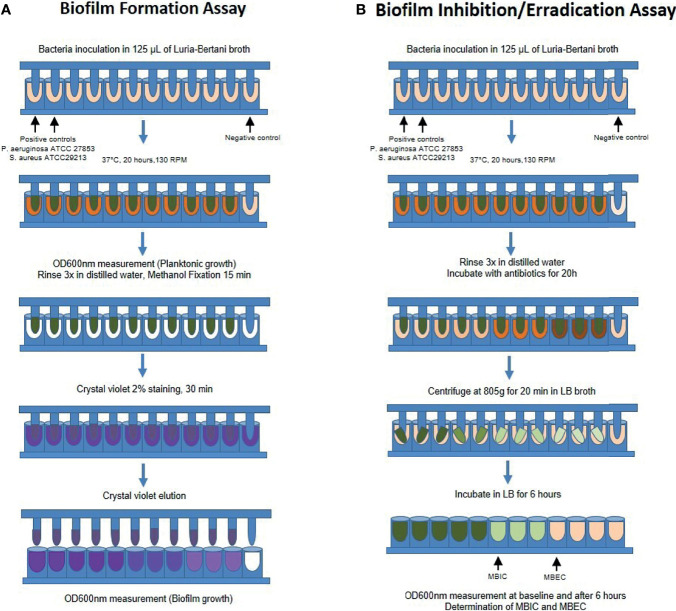
Schematic representation of the modified Calgary Biofilm Device protocol for **(A)** Biofilm formation and **(B)** Determination of the Minimal Biofilm Inhibitory (MBIC) and Eradication (MBEC) Concentrations. RPM, rotations per minute; OD, optical density; LB, Luria-Bertani; MBIC, minimal biofilm inhibitory concentration; MBEC, minimal biofilm eradication concentration.

To determine *in vitro* biofilm formation, we performed the modified Calgary biofilm assay as previously described by Moskowitz et al. (2004). Briefly, bacterial isolates were seeded for 16 hours in sterile Luria-Bertani broth at 37°C in a shaking incubator at 130 RPM (Shaking incubator SI-300, Lab Companion - Seul, South Korea), until reaching the log phase of growth. The absorbance was taken in a spectrophotometer (600 nm wavelength, BioPhotometer plus, Eppendorf – Hamburg, Germany), and samples were diluted in sterile LB broth to reach an optical density of 0.1, and eventually resuspended to 1:100 in LB medium. After dilution, 125 µL of each sample was seeded in quadruplicate in a 96-well Calgary Biofilm Device, containing a 96-well plate (Nalgene Nunc International, Rochester, NY) and a corresponding 96-peg lid (Nunc TSP system lid, catalog#445497), and incubated at 37˚C for 20 hours. After incubation, the 96-peg lid was gently rinsed 3x with sterile water to remove planktonic bacteria and fixed with 125 µL methanol for 15 minutes. After fixation, the pegs were dried at room temperature for 20 minutes and then were submerged into 160 µL of 2% crystal violet (Sigma – HT90132) for 30 minutes to stain biofilms adherent to the pegs. The lids were then rinsed 3x with sterile water and dried for 45 minutes. Finally, the crystal violet staining the pegs were eluted in 175 µL of 33% glacial acetic acid, and the plates were read in a 600 nm optical density spectrophotometer (SpectraMax M3 spectrophotometer, Molecular Devices Corporation) using the software SoftMax Pro 6.2.1 (Molecular Devices Corporation) (**Figure 1A**).

To determine the biofilm susceptibility to antibiotics [minimal biofilm inhibitory concentration (MBIC) and minimal biofilm eradication concentration (MBEC)], we performed similar steps as described previously using the Calgary Biofilm Device (CBD) in quadruplicate (Moskowitz et al., 2004; Macià et al., 2014). In brief, after biofilm formation, the 96-peg lids were rinsed in distilled sterile water and incubated in 96-well plates containing LB media with different antibiotics (amoxicillin, amoxicillin/clavulanic acid, clarithromycin, or levofloxacin) in increasing concentration 2^n^ up to 512 µg/mL. After incubation for 20 hours, the lids were rinsed 3x in sterile water and placed in a 96-well plate containing LB without antibiotics (recovery plate). The recovery plate was centrifuged at 805*g* for 20 minutes at room temperature to retrieve biofilms and incubated at 37°C for 6 hours. The OD600 of the recovery plate was read before and after incubation. As described elsewhere (Moskowitz et al., 2004; CLSI, 2018), MBIC was considered when the lowest antibiotic concentration led to a difference of OD ≤ 10% relative to the positive controls, representing a 1 log difference in growth after 6 hours of incubation. MBEC was considered when the lowest concentration of antibiotic led to a final OD similar to the negative control (LB only), corresponding to the eradication of 99.9% of bacterial biofilms recovered from the pegs (**Figure 1B**). Both MBIC and MBEC values were chosen when we observed a consistent result in at least 3 out of the 4 replicates.

For the biofilm assays, we used the strains of *P. aeruginosa* ATCC 27853 and *S. Aureus* ATCC 29213 as a positive control for biofilm-forming bacteria (Macià et al., 2014) and sterile LB as the negative control. The cut-off OD600 value to determine biofilm-forming bacteria was any mean higher than the two standard-deviation of the negative controls. To verify and validate this criterion established for biofilm formation, we performed a random selection of pegs that presented low or high OD600 values, respectively considered negative and positive biofilm-forming samples, and processed these samples for scanning electron microscopy analysis. After similar processing as previously described, the pegs were fixed in 1% osmium tetroxide for 2 hours at 4°C, rinsed in phosphate buffer 0.1M, and dehydrated in increasing ethanol concentrations up to 100%. Samples were then dehydrated by the critical point of CO_2_ method (*Critical Point Dryer* CPD 030, Bal-Tec, Schalksmühle, Germany), sputter-coated with gold (Sputter Coater SPC 050, Bal-Tec, Schalksmühle, Germany) and analyzed in the scanning electron microscope (JSM6610LV, JEOL, Tokyo, Japan) at 20 kV. Representative images were captured and saved as TIFF.

### Statistical Analysis

MIC, MBIC, and MBEC values were expressed in µg/mL. The classification as susceptible or resistant planktonic bacteria followed the Clinical Laboratory Standards Institute (CSLI) guidelines (CLSI, 2018). When values of MBIC or MBEC were undetermined, such as “higher than” or “lower than”, the highest or the lowest determined value was considered for analysis, respectively. We used the Mann-Whitney test to compare antibiofilm concentrations (MBIC/MBEC) between resistant versus susceptible planktonic bacteria, with a level of significance set at 5%.

## Results

### Demographic Data of Patients

Among the 25 patients included, the majority were female (n=19, 76%), presented CRS with nasal polyps (n=17, 68%), and had undergone prior sinus surgery (n=23, 92%), with a mean age of 43 years-old (21-68 years, SD=14). Eight patients were asthmatic (32%), 3 had aspirin intolerance, and 2 were smokers.

### Microbiological Profile

Middle meatus swabs from 25 patients with AECRS yielded 30 bacterial isolates, with a majority prevalence of 60% of *S. aureus* (18/30). Other gram-positive bacteria, such as *S. epidermidis* (n=2), *S. pneumoniae* (n=2), and *S. pyogenes* (n=1), appeared in lower frequencies. Seven gram-negative bacteria were identified (23%, 7/30), including *P. aeruginosa* (n=2), *Proteus sp* (n=2), *Citrobacter* (n=1), *Klebsiella* (n=1), and *Enterobacter* (n=1).

**Figure 2:**
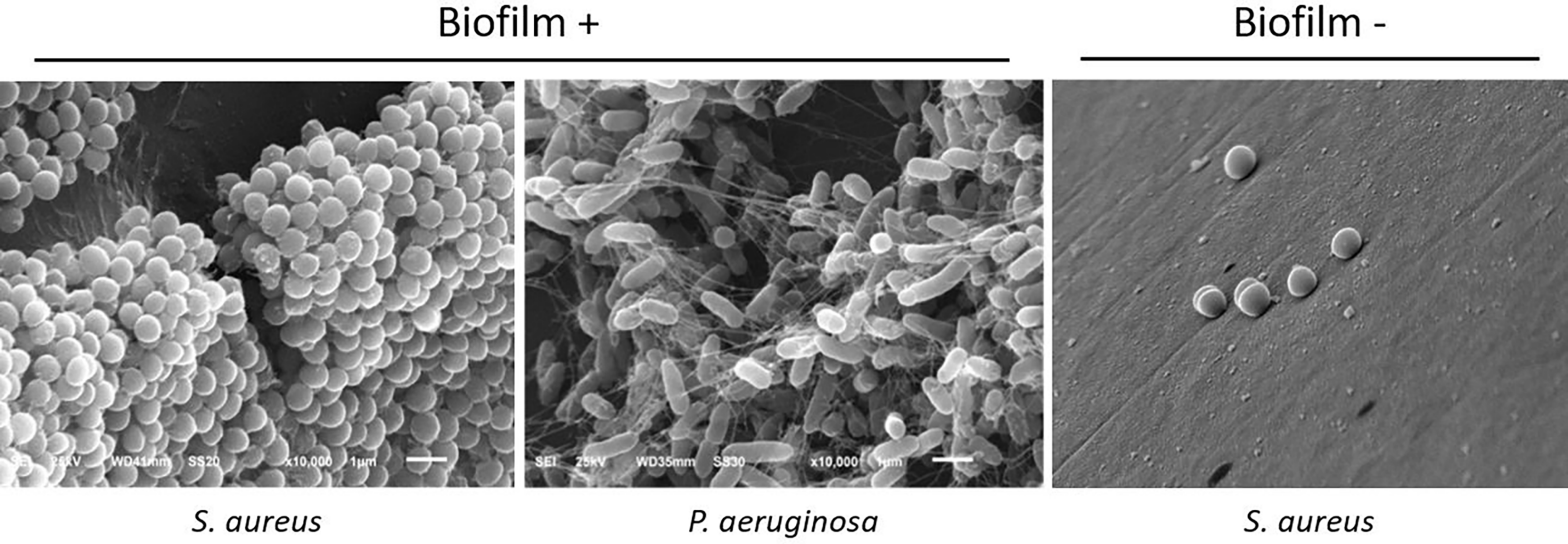
Representative scanning electron microscopy photographs of biofilm-forming and non-biofilm-forming bacteria, showing typical features of biofilms: bacterial organization in a 3D structure, adherence to the surface, and presence of extracellular matrix. 10.000x magnification.

Among bacterial isolates, 76.7% of bacteria (23/30) formed biofilm *in vitro*. Among Gram-positive bacteria, *S. aureus* and *S. epidermidis* formed biofilms in 89% (16 of 18) and 100% of cases (2 of 2), respectively. Two isolates of *S. aureus* and *S. pneumoniae*, as well as one *S. pyogenes* and one *Proteus sp*, did not form biofilm (**Figure 2**).

### Antimicrobial Susceptibility of Planktonic Bacteria

Among planktonic bacteria, 26.1% of samples were sensitive to penicillin, 39.3% to amoxicillin, and 65.5% to clarithromycin. For amoxicillin/clavulanic acid, oxacillin, and sulfamethoxazole-trimethoprim, the tested samples presented higher susceptibility of 82.1%, 85%, and 87%, respectively. The bacteria tested showed high levels of susceptibility to quinolones (92.6% to ciprofloxacin and 96.5% to levofloxacin). Notably, all samples tested were sensitive to gentamicin (**Tables 1**, **2**).

**Table 1:** Antimicrobial sensitive rates of planktonic bacteria obtained from patients with acute exacerbation of chronic rhinosinusitis, determined by the automated VITEK^®^ 2 system.

Antibiotic	Susceptibility rate (%, n=)
Penicillin	26.1% (6/23)
Amoxicillin	39.3% (11/28)
Clarithromycin	65.5% (19/29)
Ceftazidime	80.0% (4/5)
Amoxicillin/Clavulanic acid	82.1% (23/28)
Oxacillin	85.0% (17/20)
Sulfamethoxazole-Trimethoprim	87.0% (20/23)
Ciprofloxacin	92.6% (25/27)
Levofloxacin	96.5% (28/29)
Cefepime	100% (7/7)
Gentamicin	100% (27/27)

**Table 2:** Antimicrobial susceptibility of planktonic bacteria and their respective biofilm counterparts for 23 bacterial isolates.

Microbe	Amoxicillin			AMX/Clavulanic Acid			Clarithromycin			Levofloxacin		
	Planktonic	Biofilm		Planktonic	Biofilm		Planktonic	Biofilm		Planktonic	Biofilm	
	MIC	MBIC	MBEC	MIC	MBIC	MBEC	MIC	MBIC	MBEC	MIC	MBIC	MBEC
***S. aureus***	0.19 (S)	>512	>512	0.19 (S)	>512	>512	0.25 (S)	256	512	0.064 (S)	1	1
***S aureus***	<0.25 (S)	1	1	<2 (S)	1	1	<2 (S)	1	1	<1 (S)	1	1
***S.aureus***	>0.5 (R)	256	>512	1 (S)	2	4	0.5 (S)	1	4	0.094 (S)	1	1
***S.aureus***	>0.5 (R)	8	>512	<2 (S)	2	8	<2 (S)	1	1	<1 (S)	1	1
***S.aureus***	>0.5 (R)	128	512	<2 (S)	1	2	>8 (R)	>512	>512	<1 (S)	1	1
***S. aureus***	>0.5 (R)	256	>512	>4 (R)	2	8	>8 (R)	>512	>512	>8 (R)	2	16
***S.aureus***	>0.5 (R)	>512	>512	<2 (S)	4	8	<2 (S)	>512	>512	<1 (S)	1	1
***S.aureus***	>0.5 (R)	>512	>512	<2 (S)	8	16	>8 (R)	>512	>512	<1 (S)	1	1
***S.aureus***	>0.5 (R)	512	512	<2 (S)	32	32	<2 (S)	1	1	<1 (S)	1	1
***S.aureus***	>0.5 (R)	>512	>512	<2 (S)	256	>512	<2 (S)	64	512	<1 (S)	1	1
***S.aureus***	>0.5 (R)	512	>512	<2 (S)	4	4	>8 (R)	32	128	<1 (S)	1	1
***S. aureus***	>0.5 (R)	>512	>512	<2 (S)	1	2	<2 (S)	2	4	<1 (S)	1	1
***S. aureus***	2 (R)	256	>512	0.5 (S)	1	2	0.75 (S)	1	1	0.094 (S)	1	1
***S.aureus***	2 (R)	>512	>512	1 (S)	4	32	0.25 (I)	1	1	0.064 (S)	1	1
***S.aureus***	12 (R)	512	>512	1 (S)	1	1	0.094 (S)	1	1	0.5 (S)	1	1
***S. epidermidis***	<0.25 (S)	1	1	<2 (S)	1	1	<2 (S)	1	1	<1 (S)	1	1
***S. epidermidis***	3 (R)	32	256	0.75 (S)	1	4	0.25 (I)	1	1	0.094 (S)	1	1
***P. aeruginosa***	>32 (R)	>512	>512	>2 (R)	128	512	>8 (R)	8	512	<2 (S)	1	1
***P. aeruginosa***	>32 (R)	>512	>512	>2 (R)	256	>512	>8 (R)	16	256	<2 (S)	1	1
***Klebsiella***	>32 (R)	>512	>512	<2 (S)	2	2	<2 (S)	16	128	<1 (S)	1	1
***Proteus***	<0.25 (S)	8	>512	<2 (S)	8	>512	>8 (R)	128	256	<2 (S)	1	2
***Enterobacter***	>32 (R)	>512	>512	>2 (R)	>512	>512	>8 (R)	256	512	<2 (S)	1	256
***Citrobacter***	>32 (R)	>512	>512	<2 (S)	2	64	>8 (R)	16	128	<1 (S)	1	1

MIC, Minimum inhibitory concentration; MBIC, Minimal biofilm inhibitory concentration; MBEC, Minimal biofilm eradication concentration; AMX, Amoxicillin; R, Resistant; I, Intermediate; S, Susceptible.

### Antimicrobial Susceptibility of Bacterial Biofilms

We observed that amoxicillin presented a low capacity to inhibit or eradicate biofilms, with 78% of isolates (18/23) showing MBIC ≥ 128 µg/mL and 91% of samples (21/23) with MBEC≥ 256 µg/mL. For amoxicillin/clavulanic acid as well as for clarithromycin, MBIC, and MBEC levels were still high in a significant percentage of cases, although in lower proportions than amoxicillin alone [AMX/Clav Acid: MBIC ≥ 128 µg/mL in 22% (5/23) and MBEC ≥ 512 µg/mL in 26% (6/23); Clarithromycin: MBIC ≥ 128 µg/mL in 30% (7/23) and MBEC ≥ 128 µg/mL in 56% of cases (13/23)].

On the other hand, levofloxacin was highly effective in inhibiting and eradicating mature biofilms *in vitro*. All bacteria tested presented MBIC ≤ 2 µg/mL, and only two isolates presented MBEC > 2 µg/mL, demonstrating the high efficacy of levofloxacin in eradicating formed biofilms *in vitro* (**Table 2**).

### Correlation Between Planktonic and Biofilm Antimicrobial Resistance

Notably, all resistant planktonic bacteria produced tolerant biofilm forms for the same antibiotic (amoxicillin, amoxicillin/clavulanic acid, clarithromycin, or levofloxacin). We observed an overall concordance of the antimicrobial resistance pattern in planktonic forms vs. high tolerant biofilms in 79.3% of cases. For amoxicillin, for instance, 87% of planktonic forms were resistant, whereas 91% of biofilm-forming bacteria presented high levels of MBIC or MBEC (>512µg/mL). Amoxicillin/Clavulanic acid showed the lowest pattern of antimicrobial susceptibility concordance, as eight planktonic samples were susceptible (39.1%), whereas their respective biofilm counterparts were highly tolerant (**Table 3**).

**Table 3:** Antimicrobial susceptibility pattern concordance of planktonic and biofilm counterpart bacteria for four different antibiotics.

	Resistant bacteria		Antimicrobial susceptibility pattern concordance
	Planktonic	Biofilm Counterpart*	
Amoxicillin	82.6%	100%	82.6%
Amoxicillin/Clavulanic Acid	17.4%	60.9%	60.9%
Clarithromycin	39.1%	56.5%	82.6%
Levofloxacin	0.0%	8.7%	91.3%

*For comparison, the biofilm breakpoint was considered as the same planktonic breakpoint according to the CLSI.

**Figure 3:**
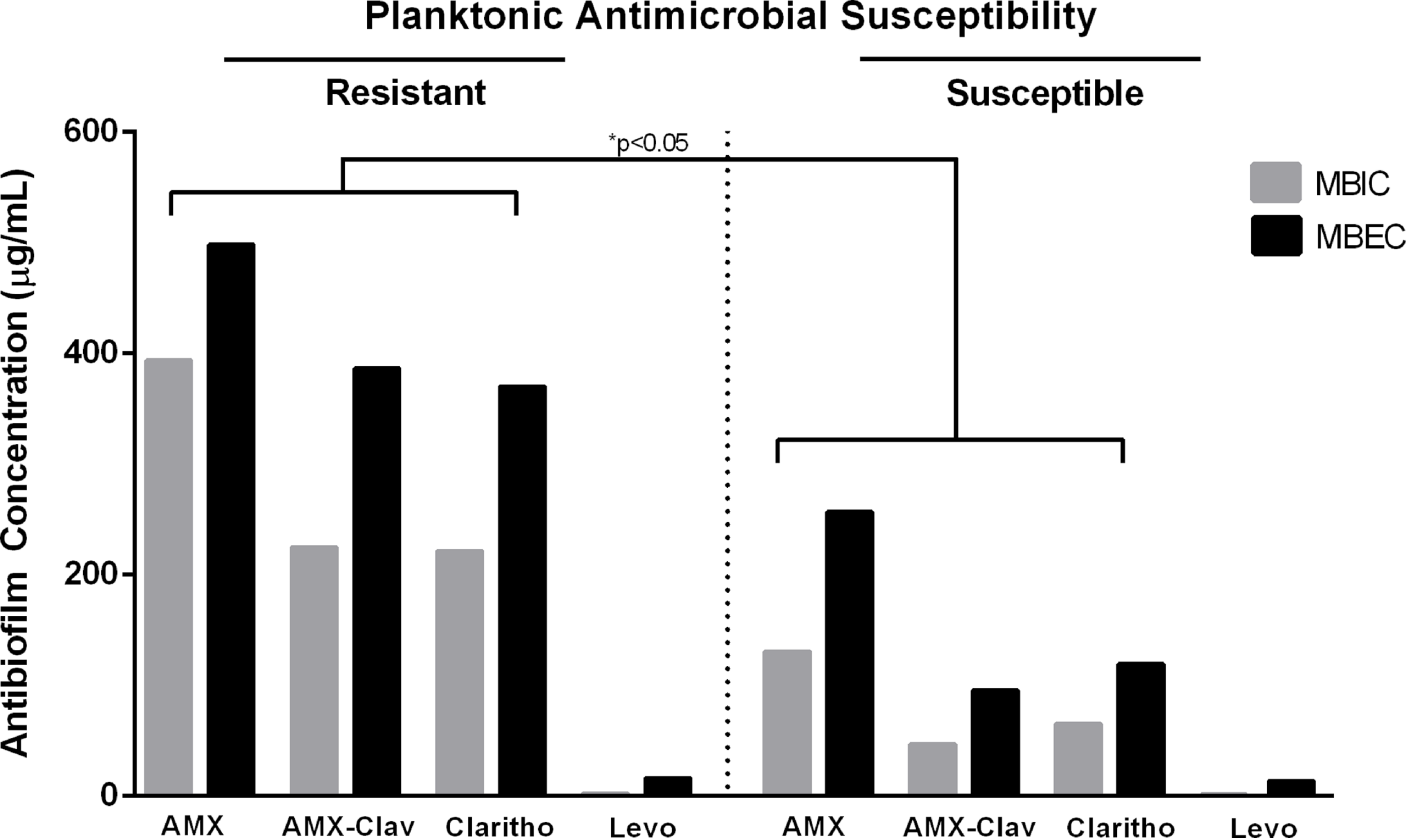
Mean values of Minimum Biofilm Inhibitory Concentration (MBIC) and Minimum Biofilm Eradication Concentration (MBEC) in resistant and susceptible planktonic bacteria for four different antibiotics, including amoxicillin (AMX), amoxicillin/clavulanic acid (AMX-Clav), Clarithromycin (Clarithro), and Levofloxacin (Levo). When MBIC and MBEC were undetermined values, such as “higher than” or “lower than,” the highest or the lowest determined value was considered for analysis, respectively. *p-values for comparisons between corresponding MBICs and MBECs of resistant vs. planktonic bacteria.

We observed higher levels of MBIC and MBEC in resistant planktonic forms than in susceptible planktonic bacteria. This relationship was notable for three antibiotics: amoxicillin (MBIC of resistant vs. susceptible planktonic bacteria= 393 vs. 130 µg/mL, *p*-value=0.016; MBEC of resistant vs. susceptible= 498 vs. 256 µg/mL, *p*-value=0.02), AMX/Clavulanic acid (MBIC of resistant vs. susceptible planktonic bacteria= 224 vs. 47 µg/mL, *p*-value=0.05; MBEC of resistant vs. susceptible= 386 vs. 95 µg/mL, *p*-value=0.0042), and clarithromycin (MBIC of resistant vs. susceptible planktonic bacteria= 221 vs. 65 µg/mL, *p*-value=0.0028; MBEC of resistant vs. susceptible= 370 vs. 119 µg/mL, *p*-value=0.005). For levofloxacin, we did not observe differences at MBIC and MBEC levels between resistant and susceptible planktonic bacteria (**Figure 3**).

## Discussion

Biofilms are a ubiquitous form in the bacterial life cycle and virtually present in all bacterial infectious diseases. Biofilm colonization of the sinonasal mucosa have been demonstrate to potentially trigger or maintain the chronic inflammation in some CRS patients, especially *S. aureus* and *P. aeruginosa* biofilms (Bendouah et al., 2006; Zhao and Wormald, 2017; Karunasagar et al., 2018; Maina et al., 2018).

The usage of antibiotics to eliminate bacterial colonization in CRS patients is still questionable, as short-term or long-term antibiotic therapy has produced controversial benefits on symptoms or endoscopic scores, even for acute exacerbations of CRS (Sabino et al., 2017; Yan et al., 2018; Wu et al., 2019). One hypothesis for the non-improvement in some patients is the high prevalence of resistant bacteria reported in CRS patients. In daily clinical practice, identifying susceptible or resistant bacteria relies mainly on classical culturable-dependent methods, which evaluate only planktonic bacteria and selected species according to the culture growth medium. As biofilms usually present high resistance to antibiotics relatively to their planktonic counterparts (Ceri et al., 1999; Ciofu et al., 2017; Łusiak-Szelachowska et al., 2021), we aimed to explore the antimicrobial resistance relationship of planktonic and biofilms in patients with AECRS.

When we induced biofilm formation *in vitro* of bacterial isolates from AECRS patients, we observed formation of biofilms in 76.6% of cases (23/30), similar to the reported rates obtained from CRS patients by Bozic et al. (2018) (92%, 46/50) and Bendouah et al. (2006) (74%, 23/31). In contrast, the biofilm-forming rate *in vitro* in our study is higher than the reported by other studies (15%, 24/156 by Zhang et al. (2015). and 28.6%, 44/157 by Prince et al. (2008). Biofilm formation *in vitro* may be influenced by several conditions of inducing and maturation of biofilm, including type of growth media, temperature, CO_2_ concentration, and time of incubation. Although the biofilm formation *in vitro* may not necessarily reflect the presence of biofilm *in vivo*, the study of biofilm resistance is important to understand possible associations with clinical outcomes in biofilm related diseases (Thieme et al., 2019).

Here, we tested four different antibiotics that are commonly used for sinonasal infections, such as amoxicillin for acute rhinosinusitis in regions with low resistant bacterial rates, and amoxicillin/clavulanic acid, clarithromycin, and levofloxacin, which are commonly used for CRS patients.

In our casuistic, we observed a predominance of gram-positive bacteria (76.7%), markedly of *S. aureus* (60%), with similar findings to other previous studies (Szaleniec et al., 2019; Yaniv et al., 2020). Among all planktonic bacteria tested, it is noticeable the high resistance rate found for amoxicillin (60.7%) and clarithromycin (34.5%). The addition of beta-lactamase inhibitor (AMX/Clavulanic Acid) reduces overall bacterial resistance, especially in planktonic bacteria (17.4%). It is noteworthy that amoxicillin alone presented low ability to inhibit or to eradicate mature biofilms in bacterial AECRS isolates, as opposed to amoxicillin/clavulanic acid.

For biofilm testing susceptibility, we were able to grow biofilms in 76.7% of samples. Notably, we observed high tolerance rates of the biofilm counterparts, especially for amoxicillin (100%), amoxicillin/clavulanic acid (60.9%), and clarithromycin (56.5%). Only two bacterial isolates (8.7%) presented biofilm-resistant forms for levofloxacin. In 79.3% of cases, the antimicrobial susceptibility profile of the planktonic was similar to the biofilm counterpart (i.e., resistant/resistant, susceptible/susceptible). In 20.7% of cases, the planktonic form was sensitive, whereas its biofilm counterpart was tolerant to antibiotics. Amoxicillin/clavulanic acid also presented a significant resistance rate in our casuistic (17.9%), whereas levofloxacin was the most effective in eradicating mature biofilms *in vitro*.

It is important to note that our casuistic were formed in the majority by more severe and recalcitrant CRS patients with acute exacerbation, as 92% of patients had undergone at least one sinus surgery, 68% of patients presented nasal polyps, and up to one third had asthma or aspirin intolerance. The high levels of biofilm-forming bacteria and, mainly, the high levels of planktonic and biofilm forms resistant to antibiotics found in our study might be related to the inclusion of more severe AECRS patients, potentially due to the prior exposure to antibiotics.

Bacterial biofilms can become recalcitrant to antibiotic activity due to multiple factors, including physical, metabolic, and genetic adaptative mechanisms of tolerance. Beta-lactams, for instance, have decreased diffusion through the extracellular matrix as biofilm matures; quinolones and beta-lactam lose efficacy in reduced metabolic bacteria; macrolide, quinolones, and beta-lactam may have limited antimicrobial action due to the upregulation of efflux pumps as a stress response (Moskowitz et al., 2004; Hengzhuang et al., 2011; Ciofu et al., 2017).

Determining the MIC from planktonic bacteria has been helpful to evaluate the susceptibility breakpoint and pharmacokinetics/pharmacodynamics parameters to predict therapeutic success in planktonic-associated diseases. On the other hand, corresponding breakpoints for biofilms are still not very well established. Antibiofilm parameters, such as MBIC and MBEC, have been used to predict clinical response in biofilm-associated diseases, together with MIC values (Barber et al., 2015; Cao et al., 2015; Haagensen et al., 2015). In our study, despite levofloxacin demonstrating lower levels of MIC, MBIC, and MBEC relative to other antibiotics, it does not necessarily represent that quinolone is more effective in treating AECRS patients than the other antibiotics evaluated in this study. However, the findings of this study raise the concern that planktonic and biofilm resistance is highly prevalent in AECRS, especially in more severe and recalcitrant patients, and these parameters should be considered when antibiotic is required. Finally, the importance of antibiofilm parameters in CRS patients still needs to be determined, whether they are associated with clinical outcomes. The applicability of the findings of this study requires further investigation, including a more extensive number of patients and post-treatment follow-up.

## Conclusions

In summary, our findings show that biofilm-forming bacteria from AECRS patients are prevalent, and biofilm forms are highly resistant to antibiotics compared to their planktonic counterparts. Antibiotic resistance observed in planktonic forms is a good indicator of biofilm resistance, although near 20% of susceptible planktonic bacteria can produce antibiotic tolerant biofilms.

## Data Availability Statement

The raw data supporting the conclusions of this article will be made available by the authors, without undue reservation.

## Ethics Statement

The studies involving human participants were reviewed and approved by the Institutional Research Board (HCRP) - Approval number 5238/2011. The patients/participants provided their written informed consent to participate in this study.

## Author Contributions

HS selected patients, collected samples, performed the biofilm assays, and analyzed the data. FV, DS, and WA-L selected patients, analyzed the data, and reviewed the manuscript. RM performed planktonic antimicrobial assays, analyzed the data and reviewed the manuscript. MF and CT collected samples and assisted with the laboratory assays. ET contributed to the concept of the study, selection of patients, data analysis, and writing of the manuscript. All authors contributed to the article and approved the submitted version.

## Funding

Fundação de Amparo à Pesquisa do Estado de São Paulo – FAPESP – Research Grant 2011/11764-6 and Student Scholarship 2013/04148-2. This study was financed in part by the Coordenação de Aperfeiçoamento de Pessoal de Nível Superior – Brasil (CAPES) – Finance Code 001.

## Conflict of Interest

The authors declare that the research was conducted in the absence of any commercial or financial relationships that could be construed as a potential conflict of interest.

## Publisher’s Note

All claims expressed in this article are solely those of the authors and do not necessarily represent those of their affiliated organizations, or those of the publisher, the editors and the reviewers. Any product that may be evaluated in this article, or claim that may be made by its manufacturer, is not guaranteed or endorsed by the publisher.
